# The Orphan Nuclear Receptor TLX Is an Enhancer of STAT1-Mediated Transcription and Immunity to *Toxoplasma gondii*

**DOI:** 10.1371/journal.pbio.1002200

**Published:** 2015-07-21

**Authors:** Daniel P. Beiting, Shinya Hidano, Julie E. Baggs, Jeanne M. Geskes, Qun Fang, E. John Wherry, Christopher A. Hunter, David S. Roos, Sara Cherry

**Affiliations:** 1 Department of Pathobiology, University of Pennsylvania, Philadelphia, Pennsylvania, Philadelphia, United States of America; 2 Department of Biology, University of Pennsylvania, Philadelphia, Pennsylvania, United States of America; 3 Department of Pharmacology and the Institute for Translational Medicine and Therapeutics, University of Pennsylvania School of Medicine, Philadelphia, Pennsylvania, United States of America; 4 Department of Microbiology, University of Pennsylvania School of Medicine, Philadelphia, Pennsylvania, United States of America; Washington University, UNITED STATES

## Abstract

The protozoan parasite, *Toxoplasma*, like many intracellular pathogens, suppresses interferon gamma (IFN-γ)-induced signal transducer and activator of transcription 1 (STAT1) activity. We exploited this well-defined host–pathogen interaction as the basis for a high-throughput screen, identifying nine transcription factors that enhance STAT1 function in the nucleus, including the orphan nuclear hormone receptor TLX. Expression profiling revealed that upon IFN-γ treatment TLX enhances the output of a subset of IFN-γ target genes, which we found is dependent on TLX binding at those loci. Moreover, infection of TLX deficient mice with the intracellular parasite *Toxoplasma* results in impaired production of the STAT1-dependent cytokine interleukin-12 by dendritic cells and increased parasite burden in the brain during chronic infection. These results demonstrate a previously unrecognized role for this orphan nuclear hormone receptor in regulating STAT1 signaling and host defense and reveal that STAT1 activity can be modulated in a context-specific manner by such “modifiers.”

## Abstract 2

Exploitation of the parasite *Toxoplasma gondii* identifies the host orphan nuclear hormone receptor TLX as a key enhancer of STAT1-dependent immune signaling and host defense.

## Author Summary

Immune responses are orchestrated by a diverse array of secreted ligands, yet the downstream transcriptional responses are coordinated by a relatively small set of key transcription factors, including nuclear factor kappa B (NF-κB) and signal transducers and activators of transcription (STATs). The molecular mechanisms that tailor the output of these immune signaling pathways to generate cell-, tissue-, or context-specific responses are poorly understood. In this study, we exploit a host–pathogen interaction, *Toxoplasma gondii* infection in mice, using a genetic screen to identify host factors that overcome parasite suppression of STAT1 signaling. We show that the orphan nuclear receptor TLX, a key regulator of brain development, enhances expression of a subset of STAT1-dependent genes in response to IFN-γ stimulation. Through genetic and pharmacological studies, we show that endogenous TLX function is required for triggering appropriate responses to IFN-γ in astrocytes. Moreover, we found that genetic disruption of TLX in mice impairs their ability to mount an effective immune response and control *T*. *gondii* infection in the brain. These data suggest that natural or synthetic ligands for TLX might be effective tools for modulating immune responses, particularly in the brain where TLX expression is highest.

## Introduction

Interferon gamma (IFN-γ) and STAT1 signaling play an essential role in cellular immunity, as indicated by extreme susceptibility to infection in mice and humans carrying mutant alleles for these genes and pathways [1–5]. The binding of IFN-γ to its cell surface receptor leads to phosphorylation, dimerization, and subsequent nuclear translocation of the transcription factor STAT1 [6]. Once inside the nucleus, STAT1 dimers recognize a consensus “gamma-activated sequence” (GAS) element (TTCN_3–5_GAA) in target genes and initiate a transcriptional program that is essential for resistance to a broad range of pathogens. While the core components of the IFN-γ signaling pathway, including Janus kinase 1 and 2 (JAK1, JAK2), and STAT1, have been known for nearly two decades [7,8], regulatory mechanisms dictating the specificity and strength of STAT1 activity within the nucleus are poorly understood, limiting our understanding of how IFN-γ /STAT1 signaling can be tailored or harnessed to respond to different challenges.

STAT1 activation is known to transcriptionally regulate several hundred genes [9], including pathways involved in host defense, apoptosis, and differentiation [10], but the biological output of STAT1 signaling can vary dramatically depending on the context in which activation occurs. For example, while STAT1 signaling suppresses cell proliferation during hematopoiesis [11,12], IFN-γ-induced STAT1 signaling can also drive hematopoietic stem cells to enter the cell cycle and proliferate to replace leukocytes lost during infection [13]. This apparent duality of STAT1 function also extends to additional immune cells: STAT1 signaling in macrophages activates a potent antimicrobial program and promotes antigen processing and presentation to T cells, yet STAT1 is also required for the ability of tumor-associated macrophages to suppress T cell function [14]. Similarly, while STAT1 drives T helper type 1 (T_h_1) cell development by activating the transcription factor T-bet [15], it is also required for the suppressive function of regulatory T cells [16,17]. The diversity of outcomes associated with STAT1 activation highlights the need to identify the cellular factors that modify or modulate STAT1 target selection to appropriately tailor the output of this core signaling pathway.

The intracellular protozoan parasite *Toxoplasma gondii* is a common pathogen of humans and other warm-blooded vertebrates and a valuable model for understanding IFN-γ-mediated immunity. Infection is initiated in the gastrointestinal tract and proceeds through an acute phase characterized by rapid parasite replication within hematopoietic and nonhematopoietic cells. IFN-γ signaling is critical in controlling parasite replication during this phase [18], and mice deficient in IFN-γ or STAT1 rapidly succumb to infection [19–21]. IFN-γ responses do not completely eradicate *Toxoplasma*, and parasites that evade this response differentiate into a slow-growing cyst form that persists as a latent infection in the central nervous system. During this chronic phase, IFN-γ remains essential in restricting parasites and prevents reactivation to the rapidly dividing form [22–24].

As a testament to the importance of IFN-γ and STAT1 signaling in the control of infections, many pathogens have evolved mechanisms to directly inhibit pathway components [25–28]. *Toxoplasma* can attenuate IFN-γ receptor-dependent STAT1 signaling [29–33]. We exploited this host–pathogen interaction as the basis for a high-throughput screen in order to identify host factors that when ectopically expressed could overcome the *Toxoplasma*-dependent block of STAT1 activity. This approach led to the identification and validation of nine transcription factors, including the orphan nuclear hormone receptor TLX (tailless, also known as NR2E1), which acts as a potent enhancer of STAT1 target gene expression. Furthermore, we found that TLX selectively potentiates a subset of STAT1-dependent targets, providing insight into how specific STAT1 programs can be tailored to impact immunity.

## Results

### A High-Throughput Screen for Activators of STAT1 Activity in Toxoplasma-Infected Cells

In order to identify host cell modifiers of STAT1 signaling in *Toxoplasma*-infected cells, we utilized a STAT1-responsive luciferase reporter, consisting of two tandemly repeated GAS elements able to bind STAT1 homodimers. Treatment of cells with IFN-γ leads to robust activation of the GAS luciferase reporter, but prior infection with *Toxoplasma* suppresses this activation by >5-fold (S1A Fig). This did not reflect a general impairment of host cell signaling, as parasites were unable to suppress tumor necrosis factor alpha (TNF-α) induction of an nuclear factor kappa B (NF-κB) reporter (S1B Fig). Overexpression of STAT1 failed to rescue pathway activity, suggesting that *Toxoplasma* impacts a step downstream of STAT1 stability (S1C Fig). Since STAT1 phosphorylation is required for dimerization, phospho-specific antibodies were used to further characterize pathogen suppression of the STAT pathway. Human osteosarcoma U2OS cells were infected with a low multiplicity of infection (MOI) to leave some cells uninfected, allowing us to evaluate STAT1 phosphorylation in both infected and naïve cells from the same cultures. Infection alone was not sufficient to trigger STAT1 activation (S1D Fig), whereas a 15-min stimulation with IFN-γ triggered phosphorylation of STAT1 in nearly every infected and uninfected cell (S1D Fig). STAT1 dimerization allows for nuclear import and subsequent DNA binding to induce transcription, and immunofluorescence revealed that STAT1 translocation to the nucleus is unaffected by infection (S1E Fig). Taken together, these data are consistent with previous reports [29–33], which indicate that *Toxoplasma* impairs STAT1 signaling by acting downstream in the pathway, at the level of nuclear STAT1 function (S1F Fig).

Conditions were optimized for high-throughput screening to identify genes that when ectopically expressed restored activity of the STAT1 pathway in infected cells, yielding Zʹ-factor scores > 0.5 (S2 Fig), a measure of assay robustness [34]. Pathway suppression could not be overcome by increasing the IFN-γ concentration (S2C Fig) Moreover, this STAT1 assay is 30 times more sensitive to the STAT1 homodimers triggered by IFN-γ stimulation than the STAT1/STAT2 heterodimers formed upon activation by type I interferon (S2D Fig). These data indicate that *Toxoplasma* suppression of the STAT1 pathway provides a robust, sensitive, and specific screen to identify enhancers of IFN-γ-induced STAT1-mediated transcription.

### Identification of Host Factors That Overcome Parasite Suppression of STAT1 Activity

**Fig 1:**
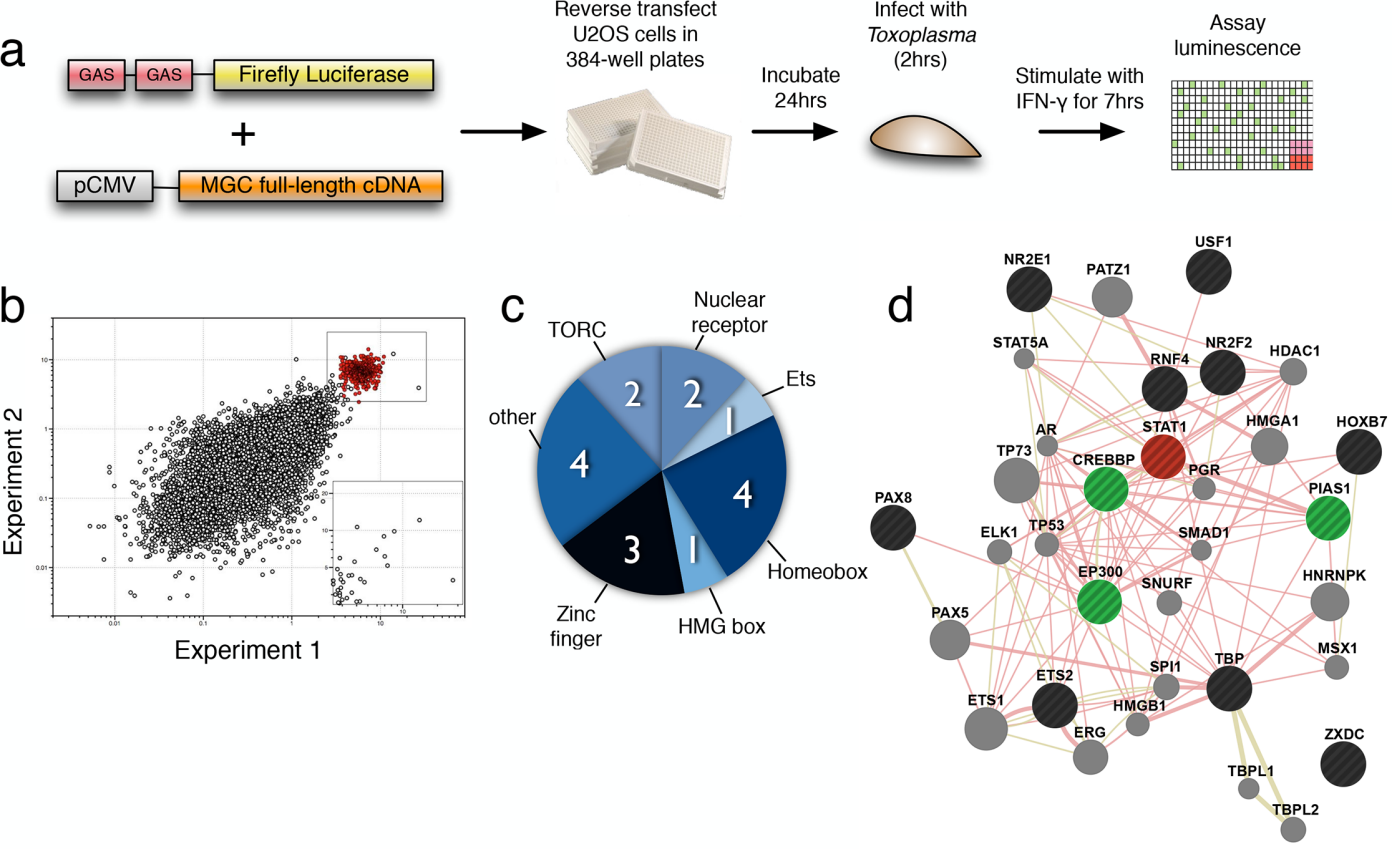
High-throughput screening identifies genes that restore function to the STAT1 pathway in *Toxoplasma*-infected cells. (A) Schematic showing workflow of STAT1 screen in *Toxoplasma*-infected cells. (B) Results of screen from experimental replicates, presented as fold change relative to infected cells transfected with an empty cDNA control vector. Uninfected cells transfected with empty cDNA controls are indicated in red, and infected cells transfected with MGC cDNA clones are in black. cDNAs that enhanced STAT1 activity ≥2.5-fold in both experiments are shown in the box in the upper right and inset (uninfected controls removed for clarity). (C) Protein domain family (Pfam)-based classification of nine validated cDNA hits that enhanced STAT1 activity ≥2.5-fold. (D) Protein–protein interaction network based on human orthologs of these hits (black) and STAT1 (red). Green indicates a network neighbor of STAT1. Data for panel B can be found in file S1 Data.

The Mammalian Gene Collection (MGC), a library of over 18,000 human and mouse full-length and sequence-validated cDNAs [35], was screened to identify genes able to restore function to the STAT1 pathway upon ectopic expression in *Toxoplasma*-infected cells (Fig 1A). The primary screen identified 32 cDNAs (17 mouse and 15 human; representing 28 genes) that enhanced STAT1 activity ≥ 2.5-fold in replicate screens of the library and had robust Z-scores ≥ 4 (Fig 1B, inset; S1 Table). Gene ontology (GO) analysis of these 32 STAT1 enhancers indicates that 21 cDNAs are involved in regulation of transcription, a 4.6-fold enrichment relative to the complete MGC library (*p*-value < 0.001). Of these 21 cDNAs, two represent orthologs of HOX5A, while two others are isoforms of mouse CRTC2. In addition, the screen identified all three ETS2 isoforms present in the library (one human and two mouse). In total, 17 unique transcriptional regulators were identified in the primary screen. Analyzing these 17 genes for Pfam domains identified motifs that defined at least six transcription factor families (Fig 1C). A network analysis (Fig 1D) reveals no previously reported direct protein–protein interactions between STAT1 (red) and the screen hits (black), but eight screen hits have been found to interact with network neighbors (green) known to directly bind STAT1, including the well-known STAT1 regulators PIAS1, CREBBP, and EP300 [36,37].

To assess whether genes identified in this screen are bona fide regulators of STAT1 activity, individual cDNA clones for the 21 putative STAT1 enhancers were sequence verified and tested for their ability to rescue STAT1 activity during parasite infection (S2 Table). Activity was also assayed in uninfected cells transfected with these cDNAs. Seventeen of the 21 cDNAs were found to enhance STAT1-dependent transcription by at least 2.5-fold in infected cells. Fifteen of these 17 cDNAs also activated transcription in uninfected cells, suggesting that the transcription factors identified through this screen represent general enhancers of IFN-γ-dependent STAT1 activation, irrespective of *Toxoplasma* infection. In order to rule out nonspecific induction of the luciferase reporter, these genes were tested for induction of a control reporter lacking the GAS elements. Six of the 17 STAT1 enhancers, including all Crtc genes and several Hox genes, induced the control reporter and therefore were not pursued further, leaving 11 cDNAs (nine genes, including two orphan nuclear hormone receptors) that specifically enhance STAT1 activity in infected and/or uninfected cells (S2 Table and S3 Fig). Finally, to rule out the possibility that these hits affected parasite fitness, we carried out additional screens of the MGC using transgenic parasites in which luciferase reports either cell invasion [38] or parasite viability [39]. These nine genes did not impact either of those assays (S2 Table), indicating that our screen identified modulators of host cell signaling, rather than direct parasite effectors, consistent with their ability to potentiate STAT1 signaling in the absence of *Toxoplasma* infection. This expands our knowledge of genes that can regulate this important signaling pathway.

### STAT1 Enhancers Exhibit Pathway Specificity

**Fig 2:**
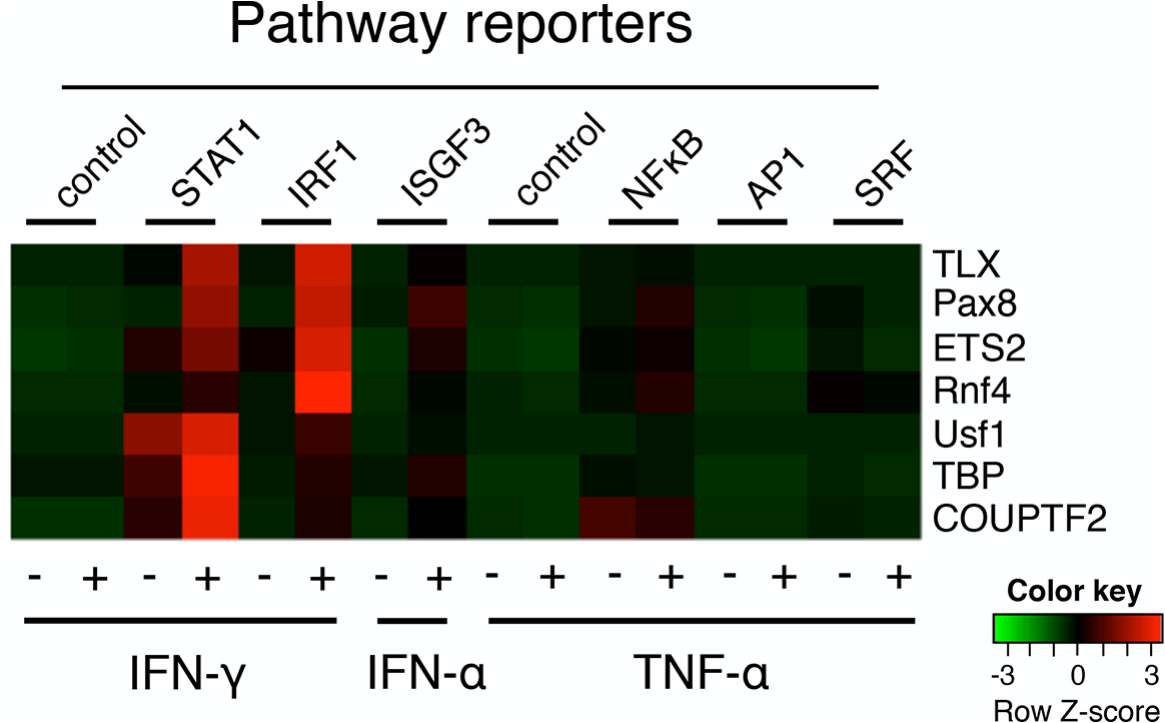
STAT1 enhancers selectively potentiate the STAT1 pathway. Heatmap of showing row Z-scores based on log2 raw luminescent values for enhancers (right) tested on various signaling pathway reporters (top), either unstimulated (-) or stimulated with the cytokines shown on bottom (+). Values are averaged from three experiments. Color scheme legend is shown.

Since the screen employed in this study was based on a reporter that responds to STAT1 homodimers, seven candidate transcription factor genes were tested against six additional pathway reporters to determine if they act specifically on the STAT1 pathway, or whether they might function as enhancers of additional immune-related signaling pathways. U2OS cells were co-transfected with candidate cDNAs along with luciferase reporter constructs under the control of either interferon response factor-1 (IRF1), NF-κB, interferon-stimulated gene factor-3 (ISGF3), serum response factor (SRF), activator protein-1 (AP1), the STAT1 homodimer reporter used in the screen (positive control), or negative control reporters (Fig 2). Cultures were then stimulated with IFN-γ to induce STAT1 and IRF1, IFN-α to induce ISGF3, or TNF-α to induce NF-κB. These particular pathway reporters and stimuli were selected because they represent well-known STAT1-dependent and STAT1-independent inflammatory pathways. Reporters for STAT1, IRF1, ISGF3, and NF-κB all responded to the appropriate stimulus with increased luminescence, while the two control reporters and AP1 and SRF reporters were not activated by any of the stimuli tested (Fig 2). All of the genes tested robustly enhanced IFN-γ-induced STAT1 homodimer activity (Fig 2). Four of the seven genes also activated IRF1 activity, consistent with the known role of IRF1 as a STAT1 target gene [40]. Moreover, these factors were not promiscuous; they displayed little activity on the other reporters (Fig 2). Taken together, these data indicate that this high-throughput screen has identified a set of transcription factors that show clear specificity for the STAT1 pathway.

### Ectopic Expression of TLX Enhances IFN-γ-Dependent Transcription of Inflammatory Mediators

We identified two orphan nuclear receptors, COUPTF2 and TLX, which exhibited strong enhancement of our STAT1 reporter (S2 Table). Because nuclear hormone receptors are well-known transcriptional regulators, acting as co-activators and/or co-repressors, and are druggable targets, and because TLX is expressed in the brain [41–43], where STAT1 activation is required to control chronic *Toxoplasma* infection [22–24], we focused our studies on TLX.

**Fig 3:**
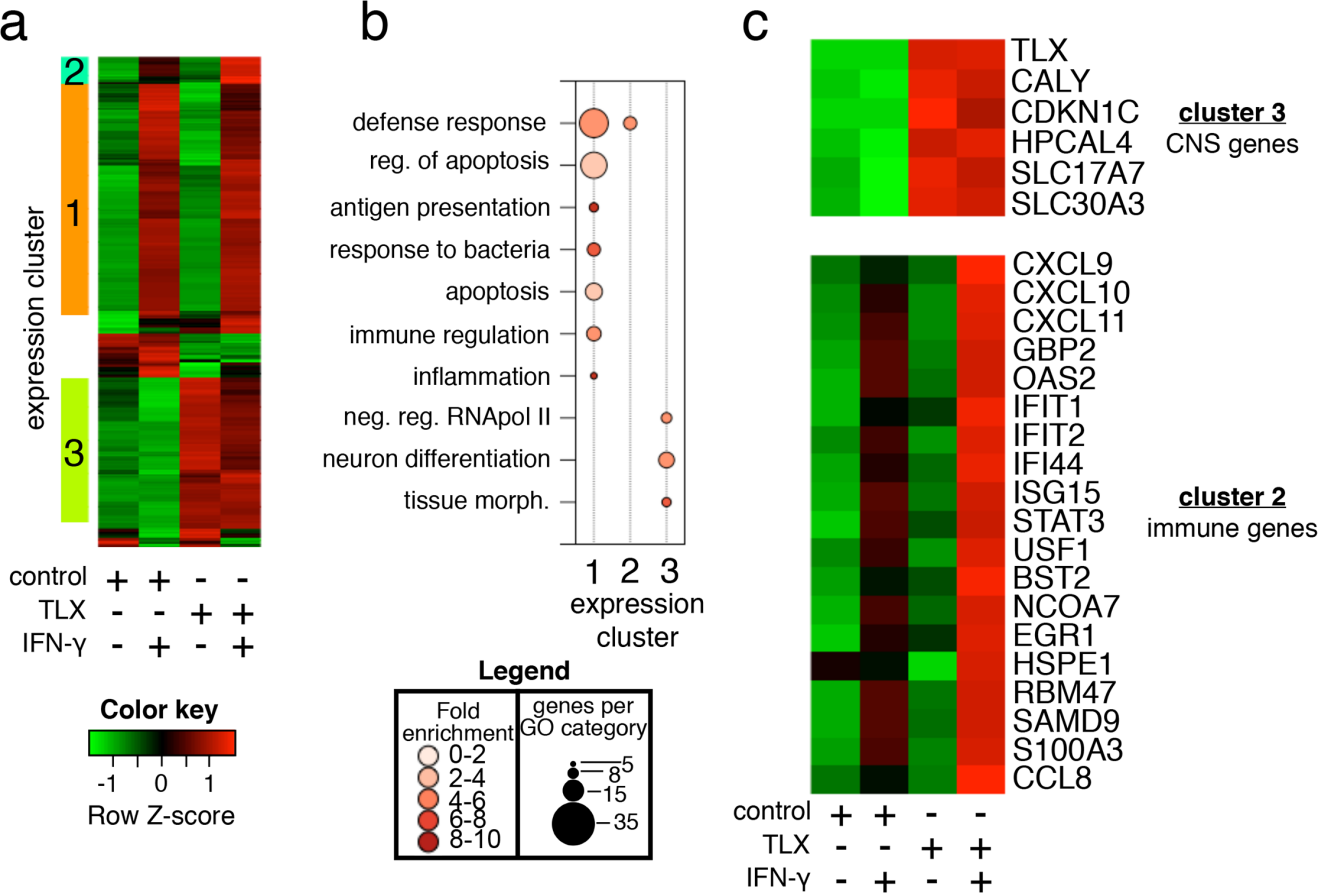
Ectopic expression of TLX potentiates a subset of IFN-γ-inducible transcripts. (A) Heatmap and hierarchical clustering analysis of 341 genes differentially regulated (≥1.5-fold, FDR ≤ 5%) plus or minus IFN-γ stimulation of cells expressing either control vector or TLX. Row Z-scores of the mean expression data for duplicate arrays are shown. (B) Bubble plot showing enriched GO terms for each of the three clusters indicated in panel A. Bubble size indicates the number of genes associated with each term, while color intensity indicates fold enrichment. (C) Heatmap showing selected genes associated with central nervous system (CNS) development and function (from cluster 3) and immune function (from cluster 2).

First, we set out to identify the spectrum of endogenous genes regulated by TLX. U2OS cells transiently expressing TLX or empty cDNA vector were untreated or stimulated with IFN-γ for 8 h and transcriptionally profiled. Hierarchical clustering of 341 differentially expressed genes (≥1.5-fold, false discovery rate (FDR) ≤ 5%) delineated at least three clusters of co-regulated transcripts (Fig 3A). GO enrichment analysis of these clusters showed that TLX overexpression enhanced transcription of 104 IFN-γ-independent genes involved in neuron differentiation and tissue morphology (Fig 3B, cluster 3), consistent with the fact that TLX is expressed in the brain, where it is an essential regulator of neurogenesis [41]. Amongst these genes are known regulators of brain physiology (Fig 3C), including brain-specific solute carriers (SLC17A7 and SLC30A3), a neuronal calcium sensor (HPCAL4), a neuron specific vesicular protein (CALY), and an essential regulator of dopamine neuron development (CDNK1C) [44], suggesting that they may represent natural targets either directly or indirectly regulated by TLX.

As expected, IFN-γ treatment of U2OS cells enhanced expression of 162 genes involved in immunity and inflammation (Fig 3A and 3B, cluster 1). Importantly, expression of 19 IFN-γ-inducible genes involved in host defense was potentiated by TLX expression (Fig 3A and 3B, cluster 2), including CXCL9, CXCL10, and CXCL11 (Fig 3C, cluster 2), all of which are well-known STAT1-dependent targets.

### TLX Depletion Impairs IFN-γ-Dependent Gene Expression

**Fig 4:**
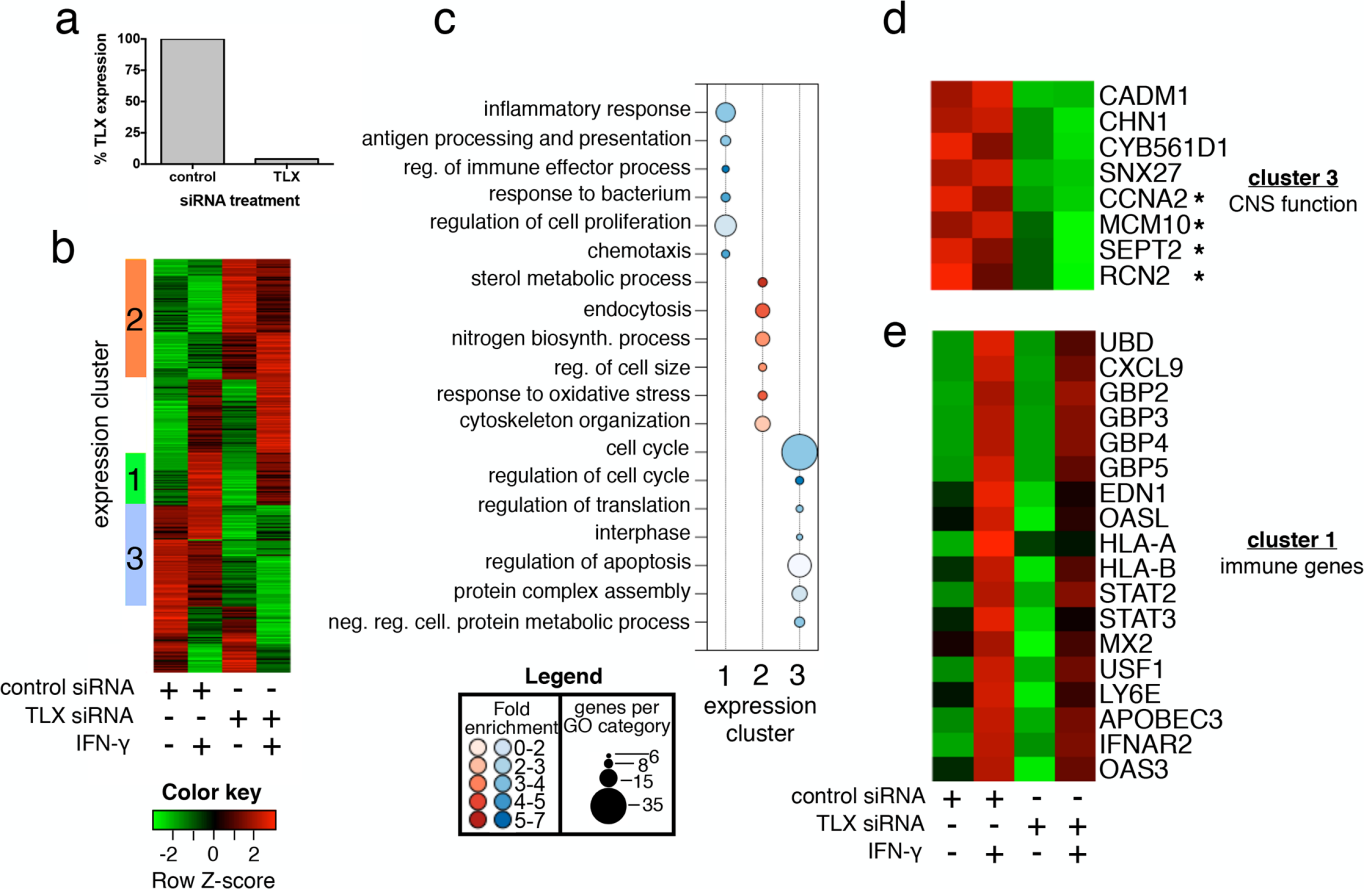
TLX depletion in astrocyte cells impairs a subset of IFN-γ-inducible targets. (A) Quantitative reverse transcription PCR (RT-qPCR) measuring TLX expression in U251 cells following treatment with either control siRNA or siRNA targeting TLX. (B) Heatmap and hierarchical clustering analysis of 1,418 genes differentially regulated (≥1.5-fold, FDR ≤ 5%) plus or minus IFN-γ stimulation of cells treated with either control siRNA or siRNA targeting TLX. Row Z-scores of the mean expression data for duplicate arrays are shown. (C) Bubble plot showing enriched GO terms for each of the three clusters indicated in panel A. Bubble size indicates number of genes associated with each term. Bubble color indicates whether genes associated with each term were up-regulated (red) or down-regulated (blue), while color intensity indicates fold enrichment. (D–E) Heatmaps showing (D) TLX-dependent genes associated with CNS development and function (from cluster 3) and (E) TLX-dependent, IFN-γ-regulated immune genes (from cluster 1). Asterisks in panel D indicate genes previously shown to be TLX dependent in the CNS [47].

Next, we sought to identify cell types that expressed high basal levels of endogenous TLX to determine the role of TLX in STAT1-dependent responses. A survey of nuclear receptor expression in the National Cancer Institute (NCI) 60 panel, a collection of cancer cells lines targeted for extensive study, including gene expression profiling [45], suggested that astrogliomas express the highest levels of TLX mRNA, with U251 cells having the highest expression [46]. Transfection of U251 cells with small interfering RNAs (siRNAs) against TLX resulted in a greater than 80% reduction in TLX transcript compared to control (Fig 4A). Next, cells transfected with siRNAs targeting TLX or with a control siRNA were either untreated or stimulated with IFN-γ for 8 h and were subject to expression profiling by microarray. Hierarchical clustering of 1,418 differentially expressed genes (≥1.5-fold, FDR ≤ 5%) delineated three clusters of co-regulated transcripts (Fig 4B). Knockdown of TLX resulted in marked repression of 352 IFN-γ-independent genes associated with cell cycle regulation (Fig 4B and 4C, cluster 3), consistent with the critical role for TLX in maintaining a proliferative state in adult neural progenitor cells [41,47]. Amongst these TLX-dependent transcripts were several genes involved in central nervous system function (Fig 4D), including a synaptic adhesion molecule (CADM1); the neuronal signal transducer, chimerin-1 (CHN1); a serotonin binding glycoprotein (GPM6B); and sorting nexin family member 27 (SNX27), a gene recently shown to regulate developmental and cognitive impairment in Down syndrome [48]. Moreover, four genes previously reported to be TLX dependent in mouse brain [47] were also identified as TLX dependent in this experiment (Fig 4D, asterisks). TLX depletion also resulted in enhanced expression of 411 genes that were enriched for sterol metabolism and endocytosis (Fig 4B and 4C, cluster 2).

U251 astroglioma cells exhibited a robust transcriptional response to IFN-γ treatment, resulting in up-regulation of a similar profile of immune defense genes as seen in U2OS cells (Fig 4B). A subset of these genes (Fig 4A, cluster 1) was TLX dependent and was enriched for GO terms relating to inflammation and antigen presentation (Fig 4C, cluster 1). These IFN-γ- and TLX-dependent genes included CXCL9 and UBD, as well as guanylate-binding proteins (GBPs) (Fig 4E). Taken together with our ectopic expression studies, these data identify TLX as a transcription factor that regulates the steady-state expression of STAT1-independent genes involved in brain function, brain development, and cell cycle while enhancing the output of a subset of IFN-γ-dependent target genes.

### TLX Potentiation of STAT1 Targets Requires Both the DNA Binding Domain and the Ligand Binding Domain

**Fig 5:**
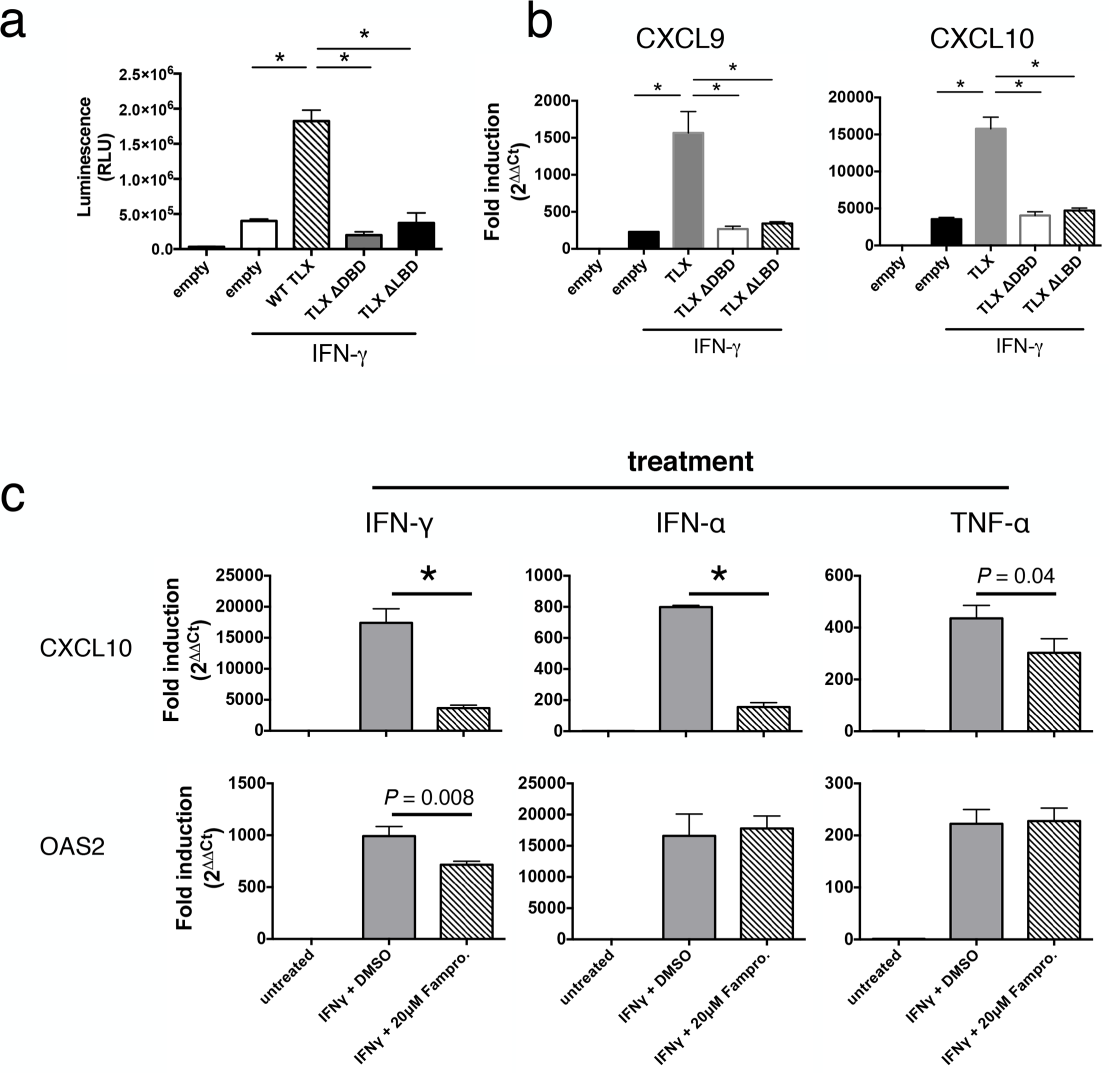
TLX potentiation of STAT1 targets requires both the DNA binding domain and the ligand binding domain. (A) STAT1 reporter activity in unstimulated or IFN-γ-stimulated U2OS cells transduced with wild-type TLX (WT) or TLX truncation mutants consisting of the DNA binding domain only (∆DBD) or the ligand binding domain only (∆LBD). (B) RT-qPCR for CXCL9 and CXCL10 expression in U2OS cells transfected with WT TLX or truncation constructs. (C) RT-qPCRfor CXCL10 and OAS2 expression in unstimulated or IFN-γ-treated U251 astrocytes pretreated with famprofazone or DMSO. Mean and standard deviation is shown for three biological replicates. * = *p* ≤ 0.01. Experiment was repeated three times with similar results. Data for panels A, B, and C can be found in file S1 Data.

Nuclear receptors can regulate gene expression either directly through DNA binding or indirectly by physically interacting with other transcription factors [49]. To determine whether DNA binding was required for TLX to regulate STAT1-dependent transcripts, U2OS cells were transfected with TLX constructs lacking either the DNA binding domain or the ligand binding domain. As expected, wild-type TLX markedly enhanced IFN-γ induction of our STAT1 reporter, and this induction was completely abrogated if only the DNA binding domain (TLX ∆DBD) or ligand binding domain (TLX ∆LBD) was used (Fig 5A). Similarly, when CXCL9 and CXCL10 expression were used as readouts of STAT1 function, both domains of TLX were also required for efficient induction of these genes following IFN-γ stimulation (Fig 5B). Taken together, these data show that TLX requires both DNA binding activity and ligand binding activity to enhance STAT1-mediated transcription.

Although endogenous ligands for TLX have not been described, a recent small-molecule screen identified famprofazone, a nonsteroidal anti-inflammatory drug, as a synthetic ligand for TLX that induces transrepression [50]. We reasoned that if endogenous levels of TLX potentiate the expression of specific IFN-γ-inducible STAT1 target genes, then famprofazone should inhibit this response. To test this, U251 astroglioma cells were pretreated with famprofazone and subsequently stimulated with IFN-γ, IFN-α, or TNF-α, and the expression of CXCL10 and OAS2 was measured by RT-qPCR (Fig 5C). CXCL10 and OAS2 were most potently induced by IFN-γ and IFN-α, respectively—consistent with their known role as canonical targets of these cytokines—whereas TNF-α had the weakest effect on both of these targets. Famprofazone treatment dramatically impaired the ability of IFN-γ and IFN-α to induce CXCL10 (Fig 5C), while a more modest impairment was observed on TNF-α induction of CXCL10. Interestingly, famprofazone also impaired induction of OAS2, but only when IFN-γ was used as the stimulus. In contrast, TNF-α and IFN-α induction of OAS2 was unaffected by famprofazone. These data suggest that TLX regulates STAT1 function in a stimulus- and target-specific manner.

### TLX Enhances Occupancy of Activated STAT1 on the CXCL9 and CXCL10 Promoters

**Fig 6:**
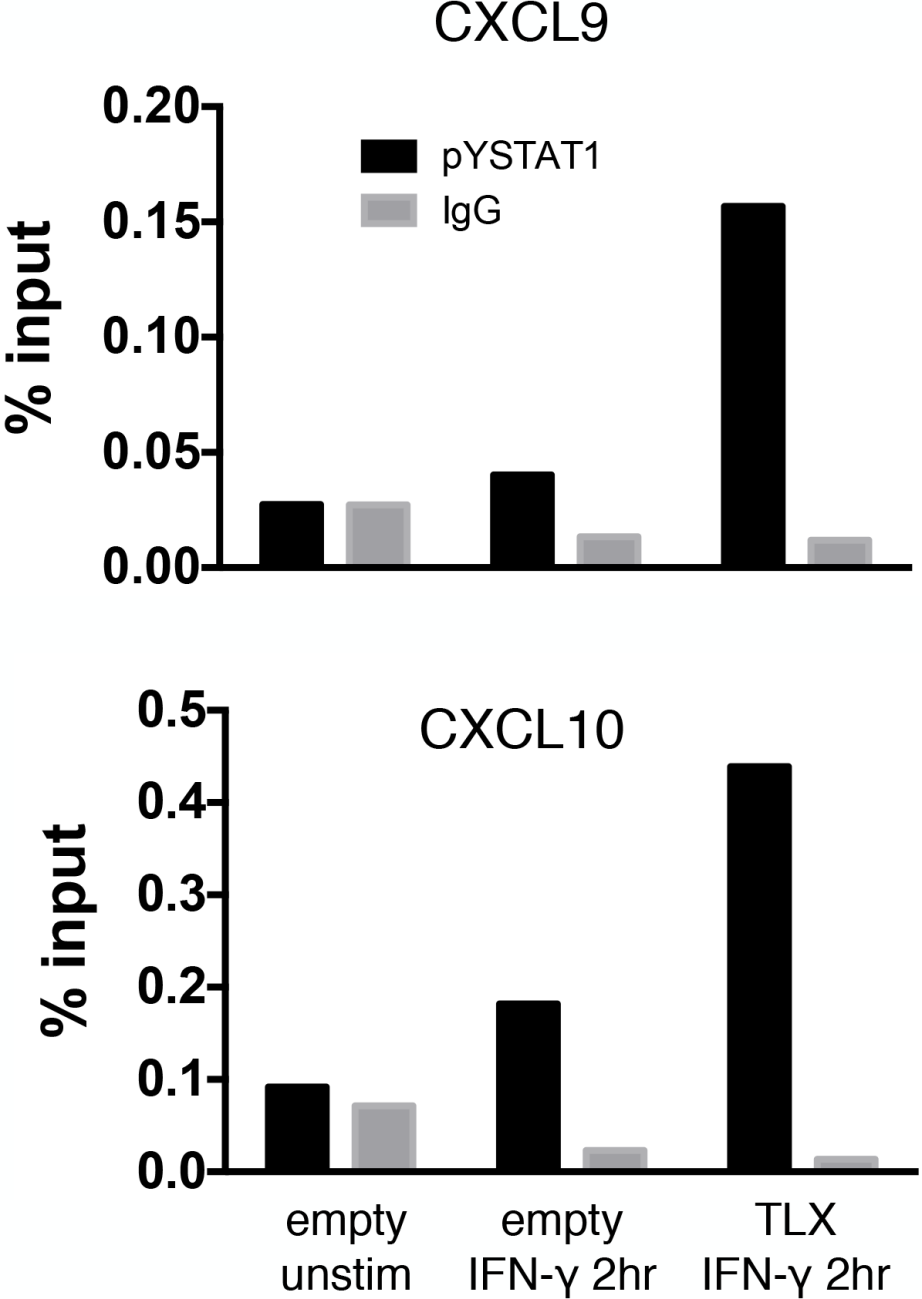
TLX expression enhances occupancy of pSTAT1 on the CXCL9 and CXCL10 promoters. ChIP with rabbit monoclonal antibody to phosphorylated STAT1 (Y701) or control rabbit IgG. U2OS cells expressing either control vector or TLX were either left untreated or stimulated with IFN-γ for 2 h. The experiment was repeated twice with similar results. Data can be found in file S1 Data.

The observation that TLX required both a DNA binding domain and a ligand binding domain for enhancement of CXCL9 and CXCL10 (Fig 5B) suggested that TLX might interact directly with the promoter of select STAT1 target genes. We hypothesized that this could increase the amount of phosphorylated STAT1 at the promoters of these genes, thereby leading to enhanced transcription. To test this hypothesis, we carried out chromatin immunoprecipitation (ChIP) in U2OS cells using antibody specific for phosphorylated STAT1, followed by quantitative PCR (qPCR) for the region of the CXCL9 and CXCL10 promoters where STAT1 is known to bind (Fig 6) [51]. Cells ectopically expressing a control vector showed increased ChIP signal at both promoters after a 2-h IFN-γ stimulation, relative to control antibody. Compared to control vector, cells expressing TLX showed a 3-fold and 2-fold increase in phosphorylated STAT1 (pSTAT1) binding at the CXCL9 and CXCL10, respectively (Fig 6). These data demonstrate that TLX can enhance transcription of IFN-γ-dependent genes by enhancing promoter occupancy of phosphorylated STAT1.

### Chronic CNS Infection with *Toxoplasma* Induces Expression of TLX in Brain

**Fig 7:**
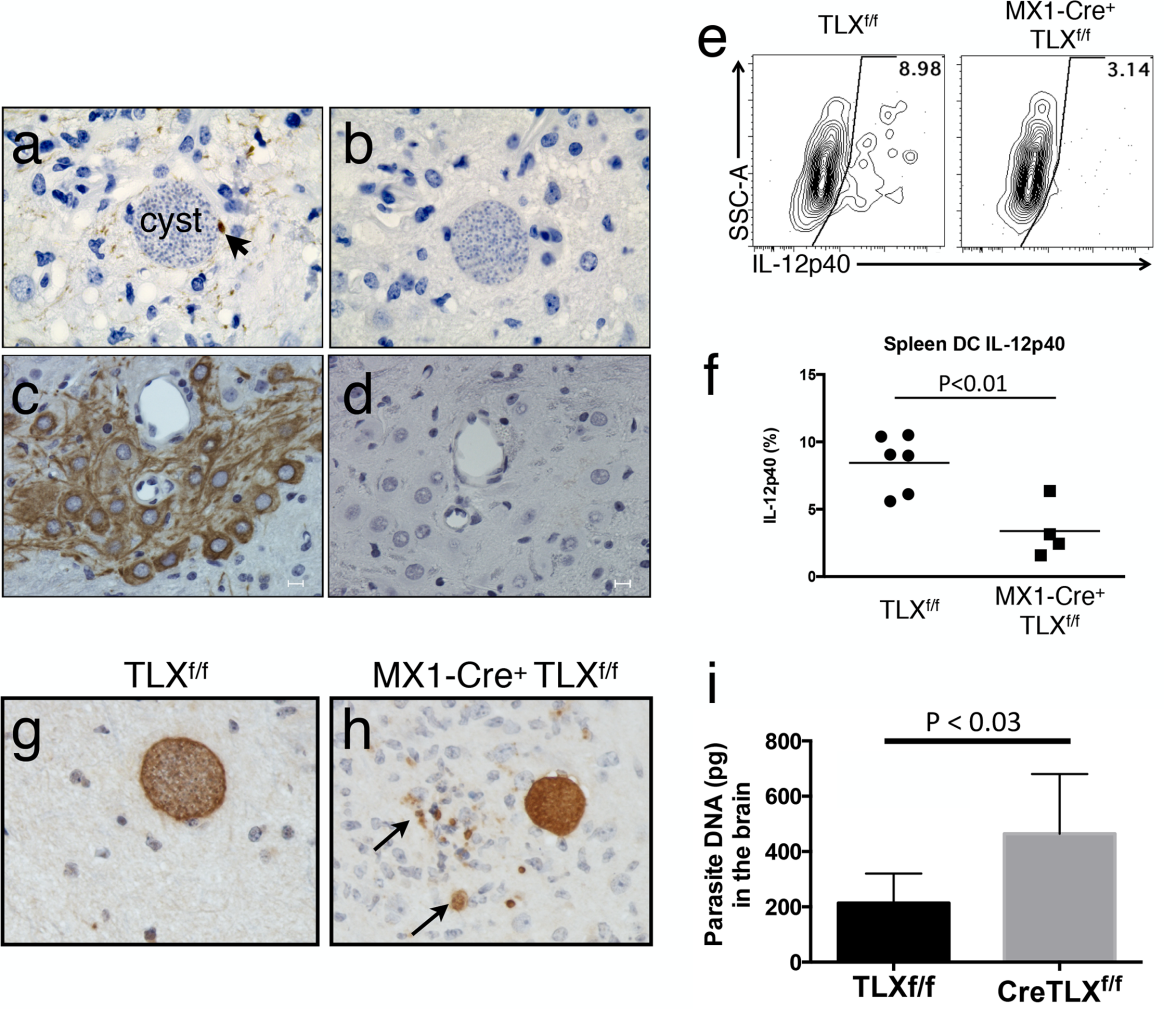
TLX is induced during *Toxoplasma* CNS infection and is required for parasite control. (A–D) Immunohistochemical staining of TLX in formalin-fixed paraffin-embedded sections from *Toxoplasma*-infected mouse brain. Sections were stained with anti-TLX (A and C) or secondary antibody as a control (B and D). (A) TLX positive cell (arrow) adjacent to *Toxoplasma* cyst. (C) Large cells with neuronal morphology are TLX positive. (E) Representative plots showing flow cytometric detection of intracellular interleukin-12 (IL-12) p40 in CD11c positive splenocytes from Mx1-cre TLX^f/f^ and TLX^f/f^ controls pretreated with Poly(I:C) prior to infection with values shown for the individual animal. (F) Graph of data from panel E for four-to-six mice per group. (G–H) Immunohistochemical detection of parasites in formalin-fixed paraffin embedded section. Free parasites are indicated by arrows. (I) qPCR-based measurement of parasite DNA in brain. Experiments were repeated two-to-three times with similar results. Data for panel F and I can be found in file S1 Data.

Although studies have explored the cell types expressing TLX in the developing and adult brain, whether TLX expression is regulated, in particular during a proinflammatory insult to the brain, is unknown. Given our finding that TLX potentiates expression of IFN-γ-inducible chemokines, we reasoned that in vivo infection with *Toxoplasma*—a potent inducer of IFN-γ production—might alter TLX expression in the brain. To test this, mice were infected and allowed to progress to chronic infection, at which point whole brains were removed, sectioned, and stained with a polyclonal antibody to TLX. Brain sections from naïve animals showed only modest, nuclear-localized staining in the granular cell layer of the dentate gyrus, a region previously described to contain TLX-positive neural progenitor stem cells (S4 Fig) [41]. In contrast, in the infected brain, numerous cell types stained positive for TLX (Fig 7), including cells with an apparent leukocyte morphology that were found near parasite cysts (Fig 7A and 7B, arrow). In addition, neurons within the cerebral cortex stained intensely for TLX (Fig 7C and 7D). These data demonstrate, for the first time, that although TLX is expressed selectively in granular layer of the dentate gyrus in the normal adult brain, CNS infection can induce TLX expression in a variety of cell types, many of which are proximal to the microbial insult.

### TLX Is Required for Optimal T_h_1 Responses and Control of Parasite Burden during Chronic Infection with *Toxoplasma*

The critical role for IFN-γ and STAT1 in restricting *Toxoplasma*, taken together with our finding that TLX is induced in the brain during infection and can regulate IFN-γ-dependent expression of molecules such as CXCL9, CXCL10, GBP4, and GBP5, prompted us to examine whether TLX impacted infection or pathogenesis. Mice carrying a floxed allele of TLX were crossed to mice expressing Cre under the control of Mx1, allowing inducible deletion of TLX [47]. Following treatment with Poly(I:C) to induce deletion, mice were rested for 2 wk and then infected with *Toxoplasma*. Animals were allowed to progress to chronic infection when parasites had established long-lived infection in the brain, at which point two key parameters of the immune response to this parasite were examined: induction of a potent T_h_1 response marked by IL-12 production and control of parasite replication.

Dendritic cells are a critical source of IL-12 during *Toxoplasma* infection, and their ability to produce this cytokine is required for parasite control [52]. In addition, IL-12 is STAT1-dependent during *Toxoplasma* infection [20], as well as in other protozoan infections [53]. Therefore, we compared dendritic cell IL-12 production in our wild-type (WT) and TLX-deficient mice. As expected, splenic dendritic cells recovered from control TLX^f/f^ infected mice produced IL-12 after in vitro treatment with brefeldin A and monensin (Fig 7E and 7F). This was associated with control of chronic infection as brain tissue stained with antisera to parasite antigens revealed intact cysts with well-formed cysts walls (Fig 7G). In contrast, Mx1Cre-TLX^f/f^ mice showed a near complete loss of IL-12 production by splenic dendritic cells (DCs) (Fig 7E and 7F) and had a marked increase in parasite burden in the brain, as demonstrated by the presence of parasites outside of cysts (Fig 7H, arrows). Furthermore, these animals presented with a higher parasite burden as assessed by qPCR for parasite DNA (Fig 7I). Taken together with our in vitro data, these results suggest that TLX plays a critical and previously unrecognized role as a STAT1 enhancer that is required for protection from *Toxoplasma* infection

## Discussion

This report presents the development and application of a host–pathogen interaction screen to interrogate a poorly understood aspect of STAT1 signaling: the regulation of STAT1 transcriptional activity in the nucleus. This strategy identified a novel set of genes that enhance STAT1 function and are highly enriched in transcription factors. The orphan nuclear receptor TLX was amongst the strongest STAT1 enhancers identified, and we have shown that TLX not only enhances the expression of endogenous STAT1 target genes following IFN-γ stimulation but is also required for control of *Toxoplasma* infection. Taken together, our data raise the possibility that TLX, as well as natural or synthetic ligands for this orphan nuclear receptor, represents an important new drug target to modulate cellular immunity and inflammation.

The use of transcription factors to elicit distinct transcriptional programs driving biological function is a common theme. This is particularly evident in the mammalian immune system, in which a small number of core transcription factors (e.g., STATs, IRFs, and NF-κBs) orchestrate diverse innate and adaptive responses. STAT1 exemplifies this plasticity in that it acts in different settings to promote both activated and suppressive macrophage and T cell function. The context-specific activity of transcription factors and signaling pathways that converge on them can be explained by a variety of mechanisms including epigenetic regulation [54], signal intensity or duration [55], and altered transcription factor complexes, since transcription factors form elaborate and dynamic multimeric complexes with co-activators/co-repressors and basal transcriptional machinery at the promoters of target genes [56]. Consistent with this notion, a recent study found that cell-type-specific responses mediated by transforming growth factor beta (TGF-β)-induced SMAD3 signaling is dictated by a small set of cell-type-specific transcription factors interacting with SMAD3 and directing its promoter binding activity [57]. Our data suggest that transcriptional modifiers also contribute to the context-specific activity of STAT1 and that TLX, in addition to its roles outside of STAT1 signaling, enhances the transcription of specific IFN-γ-regulated immune genes.

Little is known about the natural targets of TLX outside of neuronal progenitors or the involvement of TLX in immunity or as a regulator of STAT1 signaling. Our transcriptional profiling experiments reveal the spectrum of genes and functional categories regulated by TLX in two different cellular contexts. Interestingly, another nuclear receptor, the liver X receptor (LXR), in addition to its direct role in lipid metabolism, also binds to STAT1 during IFN-γ stimulation, resulting in suppression of STAT1 targets in macrophages, astrocytes, and microglial cells [58–60]. These data provide an important complement to the LXR/STAT1 interactions, by highlighting another druggable target that could drive enhanced STAT1 function in the brain, as well as in cells of the immune system. While TLX has been described as being exclusively expressed in only a select subset of cells in the brain, these data were largely derived from transgenic reporter mice [41]. Recent studies using immunohistochemistry [61] or qPCR [62] suggest that TLX may be expressed more widely than previously thought in the brain, as well as in a range of immune cell types, including CD4 and CD8 T cells, and monocytes [62].

In addition to defining TLX as a novel STAT1 enhancer, our screen revealed additional enhancers, a subset of which was previously reported to impact IFN-γ /STAT1 target genes. Upstream stimulatory factor-1 (USF1) and zinc finger X-linked duplicated family member C (ZXDC) have previously been shown to enhance IFN-γ-dependent transcription of major histocompatibility (MHC) class II [63–65]. USF1 forms a complex with STAT1 on the class II transactivator (CIITA) promoter, a well-known STAT1 target [63], and is proteolytically degraded by *Chlamydia* in order to block IFN-γ-induced transcription of STAT1 target genes [66,67]. In addition, ZXDC directly binds CIITA to enhance its function [64,65]. We also identified all three isoforms of ETS2 present in the library as STAT1 enhancers, which demonstrates the robustness of the screening assay. ETS2 physically interacts with CP300 and CBP [68]—both nuclear proteins that act as STAT1 enhancers by linking transcription factors to the basal transcriptional machinery [37]. Finally, another orphan nuclear receptor, COUPTF2, was identified in our screen. Interestingly, both TLX and COUPTF2 have recently been shown to share high homology, particularly in the region of the ligand binding domain, suggesting a functional relatedness.

While the genetic screen described in this report capitalizes on pathogen suppression of IFN-γ responses, a characteristic of a wide range of host–microbe interactions, these results highlight the potential of functional genomic screens to identify regulators of immune signaling pathways more broadly, representing a novel approach for the systematic identification of genes that modulate transcription factor activity. In addition, the data presented here specifically highlight the previously unrecognized role of the orphan nuclear receptor TLX in regulating STAT1 activity. Just as the glucocorticoid receptor and estrogen receptor are drug targets in inflammation and cancer, our data suggest that ligands for TLX may constitute new therapeutic targets to modulate inflammation and host defense in the brain.

## Materials and Methods

### Parasites, Cells, and Mice

RH strain *Toxoplasma* were maintained by serial passage in human foreskin fibroblast monolayers as described previously [69]. Me49 cysts were obtained from either Swiss Webster or CBA donor mice, enumerated by light microscopy, and used to infect mice by intraperitoneal injection of 20 cysts. TLX^f/f^ mice [47] were a kind gift from Dr. Ron Evans (The Salk Institute) and were crossed to Mx1-Cre mice to create TLX^f/f^ Mx1-Cre mice, allowing inducible deletion of TLX. TLX was deleted using 200 μg/mouse of Poly(I:C) (Imgenex) administered intraperitoneally every 3 d (five administrations total). Animals were rested for 10 d after the final dose before being infected with 20 Me49 cysts administered intraperitoneally. The human osteosarcoma (U2OS) and astroglioma (U251) cell lines were obtained from the American Type Culture Collection (ATCC) and maintained as recommended. This study was carried out in accordance with the recommendations in the Guide for the Care and Use of Laboratory Animals of the National Institutes of Health. Protocols were approved by the Institutional Animal Care and Use (IACUC) committee of the University of Pennsylvania (animal welfare assurance number A3079-01). The University of Pennsylvania Animal Care and Use Programs are fully accredited by the Association for Assessment and Accreditation of Laboratory Animal Care International (AAALAC).

### High-Throughput STAT1 Screen of the MGC

The MGC is a publically available cDNA library of complete open reading frames from the mouse and human genome [35]. cDNAs are packaged in a cytomegalovirus (CMV) promoter-based overexpression vector (sport6 vector, Invitrogen). Screening of MGC v2 was carried out in 384-well luminescence CulturPlates (Perkin Elmer) prespotted with 40 ng/well of each cDNA clone [70]. U2OS cells (7500/well) were reverse transfected for 24 h with MGC cDNA (or empty sport6 vector for control wells) and the STAT1 luciferase reporter (40 ng/well; Panomics), using Fugene 6 transfection reagent (0.24 ul/well; Roche). Cells were infected with a 10:1 ratio of RH strain *Toxoplasma* for 2 h and subsequently stimulated with recombinant mouse IFN-γ (Peprotech) for 7 h. Plates were assayed by adding BriteLite luciferase reagent (PerkinElmer) and measuring luminescence on an Analyst HT (Molecular Devices) set for 0.1-s integration time. The entire MGC library was screened in duplicate. Cells, transfection reagent, and cytokines were dispensed to each plate using a Matrix WellMate (Thermo Scientific). To minimize evaporation and edge effects during incubation, plates were covered with metal lids with a rubber gasket.

### Transcriptional Profiling

For whole-genome expression microarray, RNA was isolated using the RNeasy Plus kit (Qiagen). Biotin labeled complementary RNA (cRNA) was generated using the Illumina TotalPrep RNA amplification kit. Total RNA and cRNA quality were assessed by Bioanalyzer (Agilent). Illumina HumanHT-12 version-4 expression beadchips were hybridized with cRNA from two biological replicates per condition and scanned on an Illumina BeadStation 500GX. Scanned images were converted to raw expression values using GenomeStudio v1.8 software (Illumina). Data analysis was carried out using the statistical computing environment, R (v3.0.2), the Bioconductor suite of packages for R, and RStudio (v0.97). Raw data were background subtracted, variance stabilized, and normalized by robust spline normalization using the Lumi package [71]. Differentially expressed genes were identified by linear modeling and Bayesian statistics using the Limma package [72,73]. Probes sets that were differentially regulated (≥1.5 fold, FDR ≦ 5%; after controlling for multiple testing using the Benjamini-Hochberg method [74,75]) were used for hierarchical clustering and heatmap generation in R. Clusters of co-regulated genes were identified by Pearson correlation using the hclust function of the stats package in R. Data have been deposited on the Gene Expression Omnibus (GEO) database for public access (GSE55751).

### Functional Enrichment and Network Analysis of Screen and Microarray Data

GO enrichment analysis was carried out using the Database for Visualization and Integrative Discovery (DAVID) [76,77]. Enrichment of GO terms was defined relative to the complete MGC library or the whole human transcriptome for screen data and microarray data, respectively. Protein domains were identified using Pfam [78]. Network analysis of protein–protein interactions among human orthologs of the 17 nonredundant, putative transcription factors and STAT1 was carried out using Cytoscape v2.6.3 [79,80] and the Michigan Molecular Interaction (MiMI) plug-in [81]. MiMI was queried to find all “nearest neighbor” genes shared by at least two of the 17 query genes. Zʹ-factor analysis of STAT1 reporter assay was used to optimize conditions for screen [34]. A modified robust Z-score was calculated for each cDNA as described previously [82]. All experiments were repeated two-to-four times. Means and standard deviations were calculated from biological replicates. Significance was determined using a Student’s *t* test. Statistical analysis and data visualization were performed with GraphPad Prism 4 and DataGraph 2.3 (Visual Data Tools).

### Flow Cytometry, Intracellular Cytokine Detection, and Phospho-STAT1 Staining

At 4–8 wk postinfection, infected mice were perfused with 40 ml of ice-cold PBS to remove peripheral blood. For splenocyte preparation, spleens were dissected, dissociated, and subjected to hypotonic red blood cell lysis to generate a single cell suspension that was used for ex vivo cytokine analysis, splenocytes were stimulated for 4 h with brefeldin A and monensin, and then cells were rinsed, stained for surface markers with CD3-Pacific Blue, CD19-Pacific Blue, B220-Pacific Blue, CD11b-APC, and CD11c-PE-Cy7 at 4°C, and fixed with 4% PFA in PBS for 10 min at RT. Intracellular IL-12 staining with IL-12p40-PE was detected by staining in FACS buffer containing 0.5% saponin (Sigma, St. Louis, Missouri). DCs were identified as Dump (CD3, CD19, NK1.1)^-^, CD11c^+^. Data were collected on a BD LSRFortessa cell analyzer (BD Bioscience) and analyzed using FlowJo software (TreeStar, Ashland, Oregon). Antibodies were purchased from BD Biosciences (San Jose, California) and eBioscience (San Diego, California). For detection of phospho-STAT1, U2OS cells were infected for 2 h with *Toxoplasma* parasites engineered to express tdTomato and subsequently stimulated with 10 ng/ml of rIFN-γ (Peprotech). At various times poststimulation, cells were trypsinized (for flow cytometry only), fixed with 2% formaldehyde, permeabilized 10 min with cold methanol at 4°C, and stained with Alexa-488-conjugated monoclonal antibody specific for phosphorylated tyrosine residue 701 of STAT1 (clone 4a; BD Biosciences).

### Validation of STAT1 Enhancers and Testing on Pathway Reporter Panel

For validation of primary hits from the high-throughput screen, cDNA clones were expanded from bacterial stocks of the library, and DNA was isolated using a HiSpeed Maxi Kit (Qiagen). Each clone was sequence verified and retested in six replicate wells in 384-well format using the conditions described above for the full screen. Each clone was also tested for its ability to regulate STAT1 in uninfected cells, as well as its ability to trigger a control luciferase reporter lacking GAS elements. Additional reporters were also used for monitoring ISGF3 (Stratagene), IRF1 (ActiveMotif), NF-κB, SRF, and AP1 (Clontech), as described in the text.

### siRNA Knockdown and Small-Molecule Perturbation of TLX in the U251 Human Astroglioma Cell Line

U251 cells were plated at 200,000 cells per well in 6-well plates and allowed to adhere overnight. Cells were transfected with either 10 nM siRNA to human TLX (Qiagen; target 5ʹ-CCGGTTGATGCTAACACTCTA-3ʹ, sense 5ʹ-GGUUGAUGCUAACACUCUATT-3ʹ, and antisense 5ʹ-UAGAGUGUUAGCAUCAACCGG-3ʹ) or 10 nM siRNA to luciferase as a negative control in HiPerfect transfection reagent (Qiagen) and incubated for 72 h before stimulating with rIFN-γ (Peprotech) for 8 h. Total RNA was isolated and used for either qPCR or expression profiling by microarray as described below. For inhibitor experiments, U251 cells were treated with 20 μM famprofazone (Santa Cruz Biotechnology), for 4 h before stimulation with 10 ng/ml rIFN-γ. RNA was isolated 8 h poststimulation and used for qPCR.

### Construction of TLX Truncation Mutants

Full-length human TLX (NR2E1) cDNA (GenBank accession BC028031) from the MGC was used as a template for PCR with primers specific for the DNA binding domain (forward: 5ʹ- *ccat* ctcgag **ATG**AGCAAGCCAGCCGGA-3ʹ; reverse: 5ʹ- *ccat* tctaga **TTA**GCGGATGGTGGACGTCCG-3ʹ) and the ligand binding domain (forward: 5ʹ-*ccat* ctcgag **ATG**GAATCAGCTGCCAGACTTCTCTTCATGAG-3ʹ; reverse: 5ʹ- *ccat* tctaga **TTA**GATATCACTGGATTTGTACATATCTGAAAGCAGTC-3ʹ). Primer nucleotides in bold indicate start or stop codons; underlined are restriction sites for directional cloning, and italics indicate a 4 nt pad region permitting efficient restriction of PCR amplicon ends. PCR products were gel purified, digested overnight with XhoI and XbaI, and cloned into the pCMV-Sport6 plasmid with DNA ligase (New England Biolabs). Inserts were sequence verified, clones were cultured overnight, and DNA was isolated by maxiprep. Truncation mutant constructs were then used in STAT1 luciferase reporter and qPCR assays as described above.

### ChIP

U2OS cells were transfected in 10 cm dishes with 6 μg/dish of either Sport6-empty control plasmid or Sport6-hNR2E1 using Fugene 6 reagent. Twenty-four hours post-transfection, cells were left untreated or were stimulated with 20 ng/ml of IFN-γ and then cross-linked with 1% formaldehyde for 10 min before quenching with 125 mM Glycine for 5 min. Cells were recovered by scraping, pooled, and 20–40 million cells were pelleted and treated with cell lysis buffer (10 mM Tris pH 8.0, 10 mM NaCl, and 0.2% NP-40) for 10 min on ice. Nuclei were lysed with 50 mM Tris pH 8.0, 10 mM EDTA, and 1% SDS for 10 min at room temperature. Lysate was diluted in immunoprecipitation buffer (20 mM Tris pH 8.0, 2 mM EDTA, 150 mM NaCl, 1% Triton X-100, and 0.01% SDS) and sonicated seven times for 30 s each time, with 1 min on ice between each sonication. Lysis and dilution buffers were supplemented with protease and phosphatase inhibitors. Sonicated samples were further diluted to 3.6 ml each, and 1 ml was used for immunoprecipitation overnight at 4°C with either rabbit monoclonal antibody to pSTAT1 (Cell Signaling Technology, clone 58D6) or control rabbit IgG (Cell Signaling Technology). Bound chromatin was pulled down with protein G Dynabeads (Life Technologies) for 3 hat 4°C. Beads were washed and incubated with elution buffer (50 mM Tris pH 8.0 and 10 mM EDTA) at 65°C for 30 min. Chromatin was recovered, and cross-links were reversed by overnight incubation at 65°C. Samples were treated with RNAse A for 2 h at 37°C, followed by treatment with proteinase K for 30 min at 55°C. DNA was purified over PCR purification columns (Qiagen) and used for qPCR with primers designed based on known binding sites of STAT1 in the promoters of CXCL9 and CXCL10 [51]. CXCL9 primers were forward: 5ʹ-CAGATCCAAGGGAATTTCTGC-3ʹ and reverse: 5ʹ-TGTGCCAAAGGCTATCAGTG-3ʹ. CXCL10 primers were forward: 5ʹ-TGCCCTGACAAACTAATGAGC-3ʹ and reverse: 5ʹ-CAAGGCATATTCTGCACCAG-3ʹ.

### Immunohistochemical and Immunofluorescent Staining of TLX in Mouse Brain

Whole brain recovered from Swiss Webster mice at 4–6 wk postinfection were either snap frozen in OCT embedding media or fixed in 10% neutral buffered formalin before embedding in paraffin. For frozen tissue, 5 μm sections were cut on a Leica CM 3050 S cryostat and stained with 5 μg/ml rabbit polyclonal antibody to the N-terminus of human TLX (LifeSpan Biosciences), and bound antibody was detected using 5 ng/μl of alexa-488-conjugated goat anti-rabbit secondary. For paraffin sections, antigen retrieval was carried by microwaving slides in citrate buffer (pH 6). Images were captured on Nikon E600 microscope outfitted with a Nikon Digital Sight DS-FI1 camera (bright-field) and a Roper Scientific Photometrics CoolSnap EZ camera (fluorescence).

### Parasite Burden by Immunohistochemistry and qPCR

To detect *Toxoplasma* by immunohistochemistry, paraffin sections were deparaffinized, rehydrated, and endogenous peroxidase blocked using 0.3% H_2_O_2_ in PBS for 10 min at room temperature. Sections were blocked with 2% normal goat serum before being incubated overnight at 4°C with rabbit polyclonal antibody specific for the SAG1 protein of *Toxoplasma*. Bound antibody was detected with 1 μg/ml biotinylated goat anti-rabbit IgG using the VectaStain kit (Vector Labs), followed by DAB substrate kit (Vector Labs) according to the manufacturer’s instructions. Sections were counterstained with hematoxylin and images collected on a Nikon E600 fluorescent microscope (Nikon, Tokyo, Japan) and analyzed using NIS-Element (Nikon). Quantification of parasite DNA by qPCR was performed as previously described [83]. Briefly, 50 mg of tissue was disrupted by repeated passage through an 18 gauge needle, and DNA was isolated using the High Pure PCR template preparation kit (Roche). Real-time PCR specific for the *Toxoplasma* B1 repeat region was used to quantify the amount of parasite DNA from 500 ng of DNA purified from tissue. Samples were amplified using *Toxoplasma* B1 primers (forward: 5ʹ-TCCCCTCTGCTGGCGAAAAGT-3ʹ and reverse: 5ʹ-AGCGTTCGTGGTCAACTATCGATTG-3ʹ) and Power SYBR Green PCR Master Mix and a 7500 Fast Real-Time PCR System. A standard curve prepared from known amounts of purified *Toxoplasma* DNA was used for quantification. Analysis was performed with system software v1.3.1 (Applied Biosystems, Warrington, United Kingdom).

## Supporting Information

S1 Data Numerical data underlying panels in the figures corresponding to the datasheets as indicated on the tabs of the excel sheet are provided. Please see the Methods section for how the data was analysed to generate these numerical values.
(XLSX)

S1 Fig *Toxoplasma* suppresses IFN-γ responses downstream of STAT1 nuclear translocation. U2OS cells transduced with (A) STAT1 or (B) NF-κB responsive luciferase reporters were infected with *Toxoplasma* prior to stimulation with IFN-γ or TNF-α, respectively. (C) Ectopic expression of STAT1 (right) or empty control vector (left) does not impact parasite suppression of STAT1 pathway reporter. Asterisks indicate significant reduction in STAT1 reporter activity in infected cells (*p* < 0.001). (D) Representative dot plots of phospho-specific flow cytometric analysis of pTyr-701-STAT1 (pSTAT1) staining in *Toxoplasma-*infected U2OS cells either left unstimulated (left) or stimulated 15 min with IFN-γ (right). Numbers on the dot plot indicate the percentage of total cells in each quadrant from one experiment. (E) Immunofluorescence detection of pSTAT1 (green) in uninfected and U2OS cells infected with *Toxoplasma* (red); both samples were stimulated with IFN-γ for 15 min. Nuclear DNA was stained with DAPI (blue). (F) Model for parasite suppression of IFN-γ/STAT1 pathway. Data for panels A, B, and C can be found in file S1 Data.
(TIF)

S2 Fig Optimization of STAT1 reporter assay in *Toxoplasma*-infected cells. (A) Dose-dependent suppression of the STAT1 pathway reporter by *Toxoplasma* and (B) Z-factors calculated in this 384-well format assay. (C) Impact of IFN-γ concentration on parasite suppression of STAT1 pathway. Wedges indicate increasing dose of parasites added to cultures (MOI from 0–20, as in panel A). (D) Specificity of GAS luciferase reporter for IFN-γ versus IFN-α stimulation. Data for panels A, B, C, and D can be found in file S1 Data.
(TIF)

S3 Fig Results of secondary validation of all 21 cDNAs from S2 Table. Fold induction of the STAT1 reporter relative to an empty cDNA vector (black bars) or fold induction of the STAT1 reporter relative to a reporter lacking GAS elements (white bars) is shown for infected and uninfected U2OS cells stimulated with IFN-γ. Dotted line indicates 2.5-fold cut-off set for secondary validation (*p* < 0.05). Gray shaded boxes indicate cDNA isoforms or orthologs of the same gene. Data can be found in file S1 Data.
(TIF)

S4 Fig TLX staining in the brain of a naive mouse. Formalin-fixed, paraffin-embedded sections of the dentate gyrus of the hippocampus (upper panel, box) were examined for TLX staining (lower panel). Cells in the granular layer of the dentate gyrus stained positive for TLX (arrows). Scale bars on upper and lower panel are 100 um and 10 um, respectively.
(TIF)

S1 Table Excel spreadsheet showing results for replicate screening of a STAT1 reporter assay in *Toxoplasma-*infected U2OS cells. Raw luminescent values, as well as fold-change and robust Z-score calculations, are shown.
(XLSX)

S2 Table 17 transcription factors, represented by 21 cDNAs, that enhanced STAT1-mediated transcription in infected cells ≥2.5-fold over empty cDNA vector control (uppercase = human; lowercase = mouse). Average fold changes from replicate screens are shown for each gene for both the primary screen and the validation in U2OS cells +/- *Toxoplasma* infection. During validation, cDNAs were also tested for induction of a control luciferase reporter lacking GAS elements (“yes” indicates induction of this control reporter). Results of secondary screens to assess effects of cDNAs on parasite invasion and viability are also shown (“no” indicates no effect on parasite fitness). Asterisks to the right of gene symbols mark 11 cDNAs validated by secondary assays and screens.
(XLSX)

## Abbreviations:

AAALAC: Association for Assessment and Accreditation of Laboratory Animal Care International
API: activator protein-1
ATCC: American Type Culture Collection
ChIP: chromatin immunoprecipitation
CIITA: class II transactivator
CMV: cytomegalovirus
CNS: central nervous system
cRNA: complementary RNA
DAVID: Database for Visualization and Integrative Discovery
DC: dendritic cell
FDR: false discovery rate
GAS: gamma-activated sequence
GBP: guanylate-binding protein
GEO: Gene Expression Omnibus
GO: gene ontology
IACUC: Institutional Animal Care and Use
IFN-γ: interferon gamma
IL-12: interleukin-12
IRF1: interferon response factor-1
ISGF3: interferon-stimulated gene factor-3
JAK: Janus kinase; LXR, liver X receptor
NCI: National Cancer Institute
NF-κB: nuclear factor kappa B
MGC: Mammalian Gene Collection
MHC: major histocompatibility
MiMI: Michigan Molecular Interaction
MOI: multiplicity of infection
Pfam: protein domain family
pSTAT1: phosphorylated STAT1
qPCR: quantitative PCR
RT-qPCR: quantitative reverse transcription PCR
siRNA: small interfering RNA
SRF: serum response factor
STAT: signal transducer and activator of transcription
TGF-β: transforming growth factor beta
T_h_1: T helper type 1
TNF-α: tumor necrosis factor alpha
USF1: upstream stimulatory factor-1
WT: wild type
ZXDC: zinc finger X-linked duplicated family member C
