# Supplementary figures and images for: The Orphan Nuclear Receptor TLX Is an Enhancer of STAT1-Mediated Transcription and Immunity to Toxoplasma gondii
Source: PLoS Biol. 2015 Jul 21;13(7):e1002200. doi: 10.1371/journal.pbio.1002200 (PMC4509904; doi:10.1371/journal.pbio.1002200)

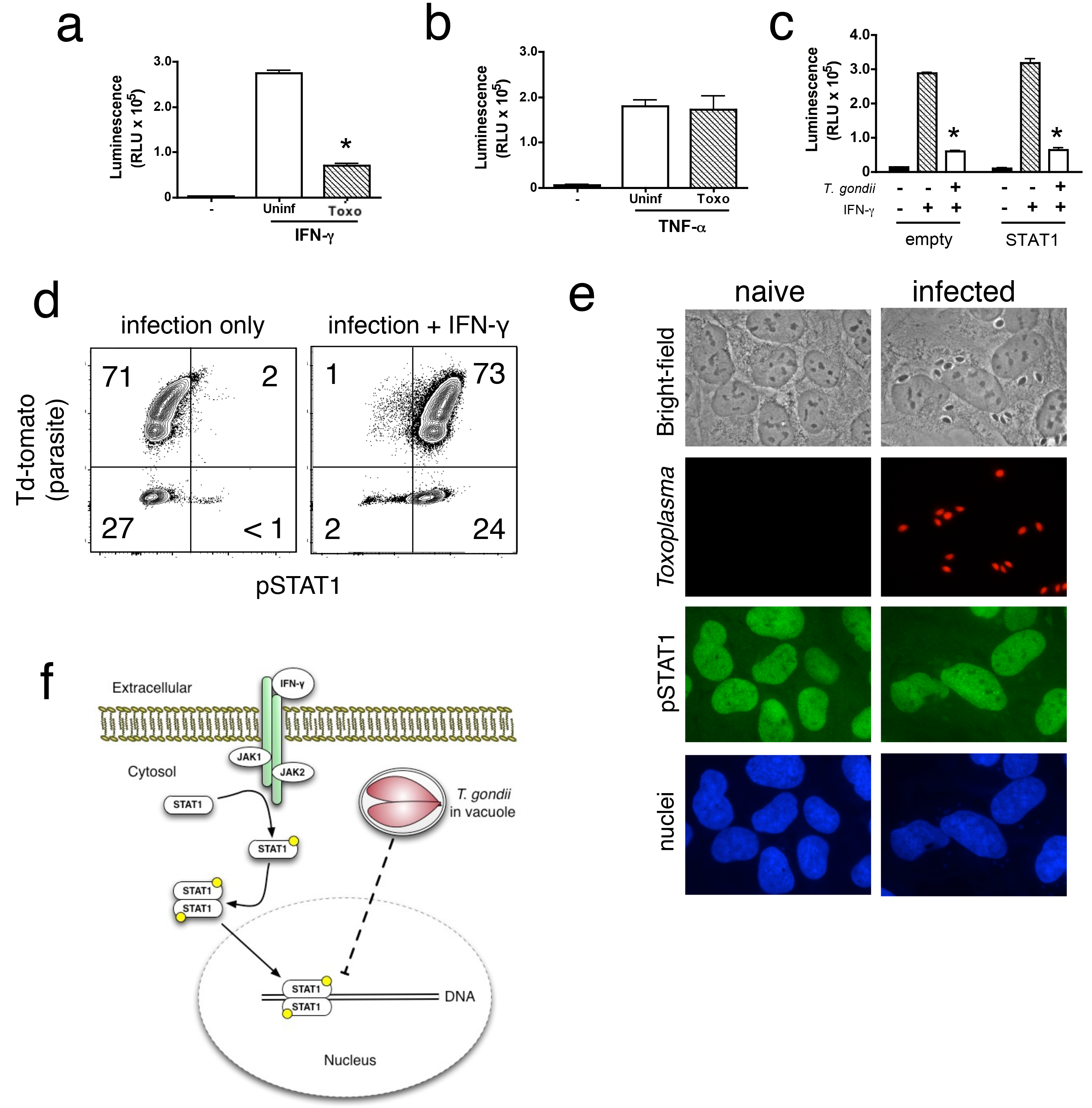

Supplement: S1 Fig — U2OS cells transduced with (A) STAT1 or (B) NF-κB responsive luciferase reporters were infected with Toxoplasma prior to stimulation with IFN-γ or TNF-α, respectively. (C) Ectopic expression of STAT1 (right) or empty control vector (left) does not impact parasite suppression of STAT1 pathway reporter. Asterisks indicate significant reduction in STAT1 reporter activity in infected cells (p < 0.001). (D) Representative dot plots of phospho-specific flow cytometric analysis of pTyr-701-STAT1 (pSTAT1) staining in Toxoplasma-infected U2OS cells either left unstimulated (left) or stimulated 15 min with IFN-γ (right). Numbers on the dot plot indicate the percentage of total cells in each quadrant from one experiment. (E) Immunofluorescence detection of pSTAT1 (green) in uninfected and U2OS cells infected with Toxoplasma (red); both samples were stimulated with IFN-γ for 15 min. Nuclear DNA was stained with DAPI (blue). (F) Model for parasite suppression of IFN-γ/STAT1 pathway. Data for panels A, B, and C can be found in file S1 Data. (TIF) [file pbio.1002200.s002.tif]

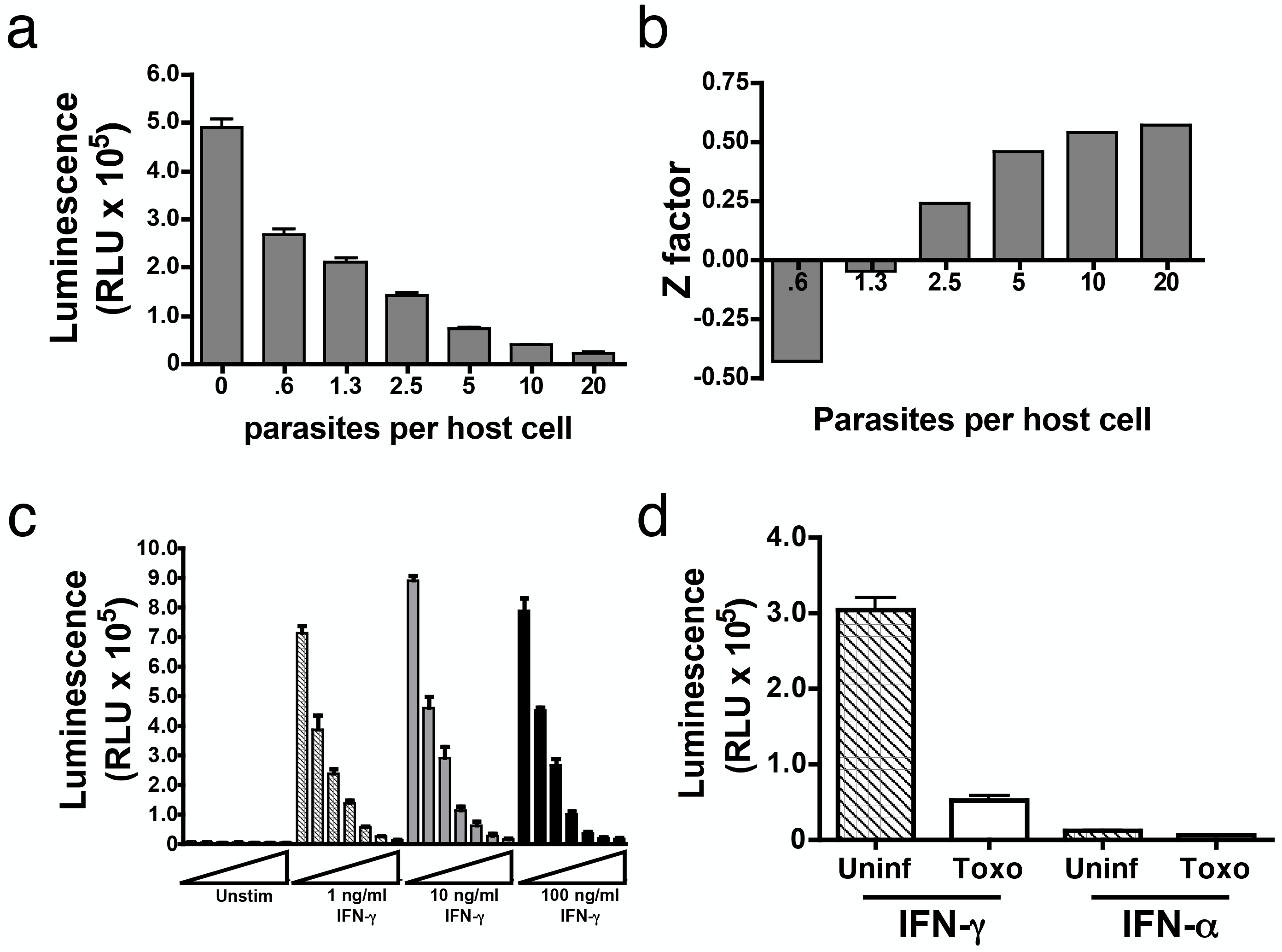

Supplement: S2 Fig — (A) Dose-dependent suppression of the STAT1 pathway reporter by Toxoplasma and (B) Z-factors calculated in this 384-well format assay. (C) Impact of IFN-γ concentration on parasite suppression of STAT1 pathway. Wedges indicate increasing dose of parasites added to cultures (MOI from 0–20, as in panel A). (D) Specificity of GAS luciferase reporter for IFN-γ versus IFN-α stimulation. Data for panels A, B, C, and D can be found in file S1 Data. (TIF) [file pbio.1002200.s003.tif]

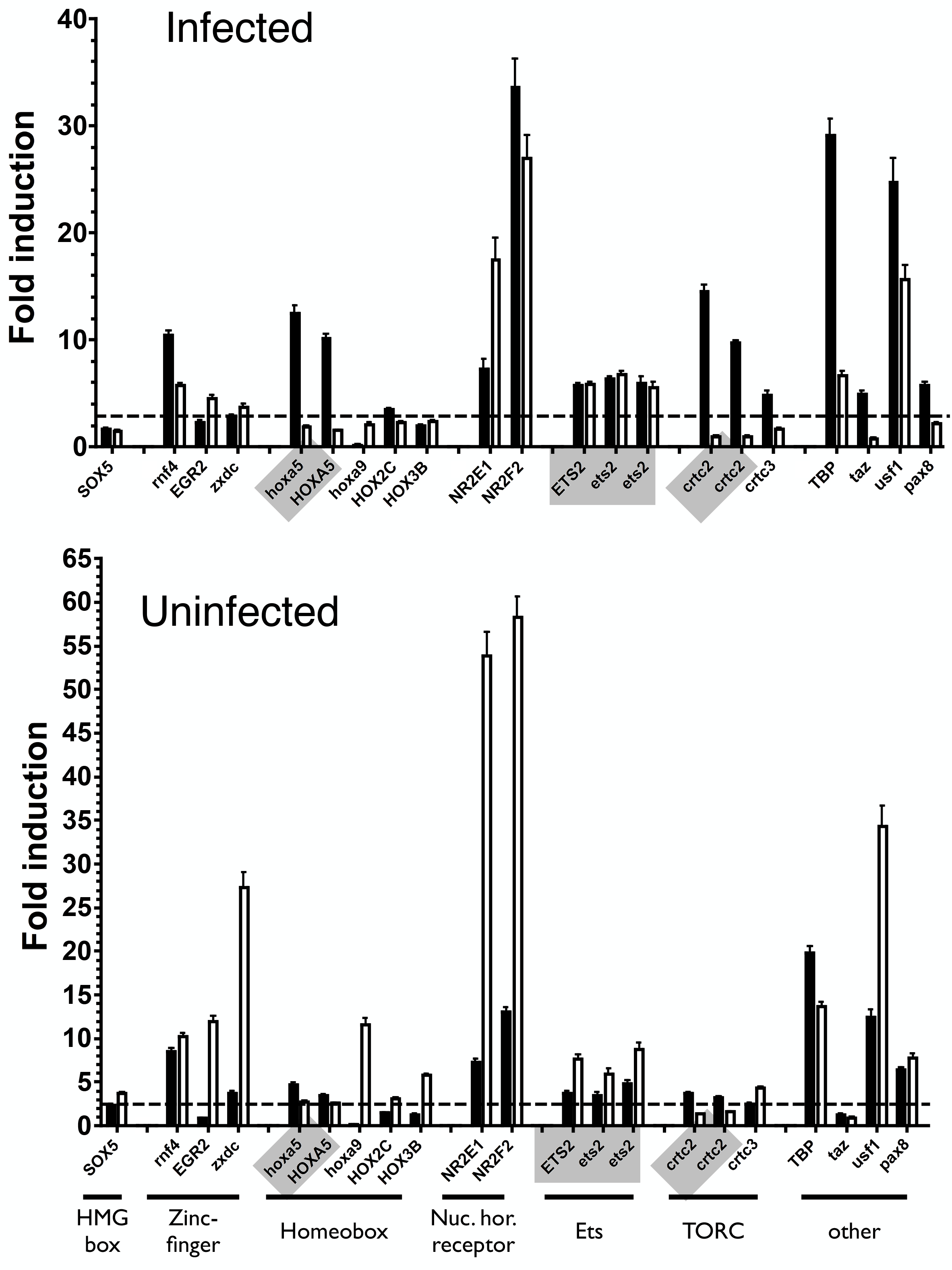

Supplement: S3 Fig — Fold induction of the STAT1 reporter relative to an empty cDNA vector (black bars) or fold induction of the STAT1 reporter relative to a reporter lacking GAS elements (white bars) is shown for infected and uninfected U2OS cells stimulated with IFN-γ. Dotted line indicates 2.5-fold cut-off set for secondary validation (p < 0.05). Gray shaded boxes indicate cDNA isoforms or orthologs of the same gene. Data can be found in file S1 Data. (TIF) [file pbio.1002200.s004.tif]

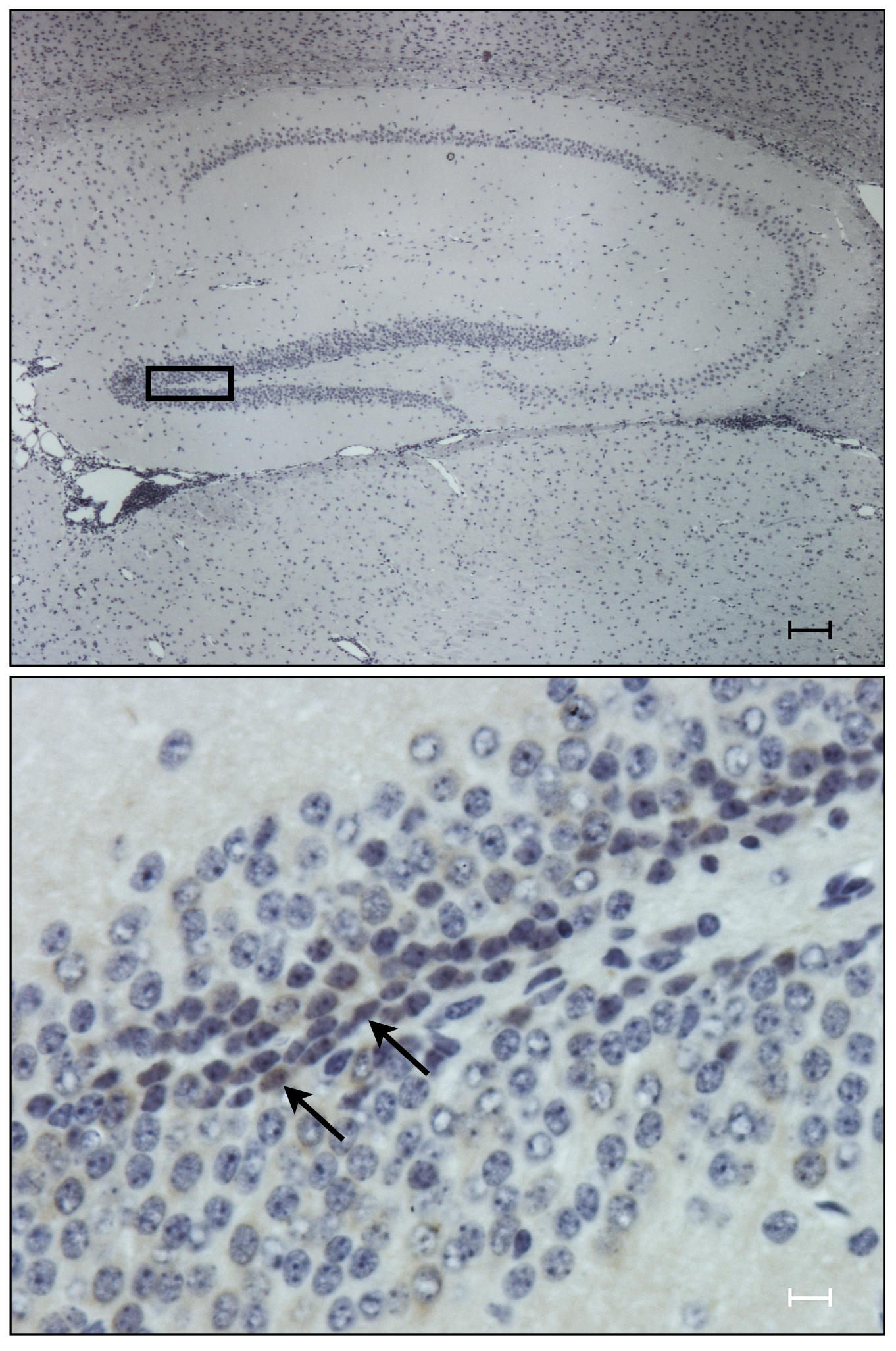

Supplement: S4 Fig — Formalin-fixed, paraffin-embedded sections of the dentate gyrus of the hippocampus (upper panel, box) were examined for TLX staining (lower panel). Cells in the granular layer of the dentate gyrus stained positive for TLX (arrows). Scale bars on upper and lower panel are 100 um and 10 um, respectively. (TIF) [file pbio.1002200.s005.tif]
